# Wavelet-Based Analysis of Soundscape Dynamics in a Riparian Woodland: The Bernate-Ticino River Park

**DOI:** 10.3390/s25237248

**Published:** 2025-11-27

**Authors:** Roberto Benocci, Giorgia Guagliumi, Andrea Potenza, Valentina Zaffaroni-Caorsi, Hector Eduardo Roman, Giovanni Zambon

**Affiliations:** 1 Department of Earth and Environmental Sciences (DISAT), University of Milano-Bicocca, Piazza della Scienza 1, 20126 Milano, Italy; g.guagliumi@campus.unimib.it (G.G.); a.potenza@campus.unimib.it (A.P.); valentina.zaffaronicaorsi@unimib.it (V.Z.-C.); giovanni.zambon@unimib.it (G.Z.); 2 Department of Physics, University of Milano-Bicocca, Piazza della Scienza 3, 20126 Milano, Italy

**Keywords:** environment sound in urban parks, eco-acoustic indices, energy-derived metrics, discrete wavelet transforms, temporal fractal analysis, Hurst exponent, biophonic inter-peak distributions

## Abstract

Passive acoustic monitoring (PAM) is a valuable tool for ecological research, but many eco-acoustic indices show inconsistent correlations with biodiversity due to methodological variability and environmental noise. We propose a complementary, physically interpretable approach using energy-derived metrics. We analyzed audio recordings from three sites near a major highway in the Ticino River Park (Milan, Italy) using 1 sec equivalent continuous sound pressure level (Leq_1s_), peak interval statistics, maximal-overlap discrete-wavelet transform (MODWT), and temporal fractal analysis. This multi-resolution type of approach enabled frequency-specific tracking of acoustic energy and temporal structure. Our results reveal site-specific differences: Site 3, the most distant from the highway, showed higher high-frequency energy and longer temporal persistence, suggesting richer biophonic activity. Site 1, the closest to the highway, displayed flatter spectral profiles and faster autocorrelation decay. Diel patterns were reflected in hourly Leq trends, while fractal analysis revealed frequency- and site-dependent acoustic memory. These automated findings were corroborated by expert annotations of bird activity and traffic. The integration of Leq_1s_, peak metrics, and wavelet decomposition offers a suitable framework for soundscape characterization, with strong potential for long-term ecoacoustic monitoring and habitat quality assessment in complex environments.

## 1. Introduction

The term soundscape was introduced to describe the interplay between the physical landscape and the ensemble of biological (biophony), geophysical (geophony), and human-made (technophony) sounds that occupy it. In recent decades, passive acoustic monitoring (PAM) has enabled the continuous, non-invasive sampling of these sonic patterns, leading to the emergence of soundscape ecology and a suite of descriptors known as eco-acoustic indices [1,2,3,4].

Widely used metrics such as Acoustic Complexity Index (ACI) [5,6,7] and Acoustic Entropy Index (H) [8,9,10] condense information on pitch, modulation, and amplitude into single-value indicators. As a result, they have been promoted as proxies for species assemblage diversity and habitat quality. Despite their potential, recent reviews have highlighted that many eco-acoustic indices show inconsistent or unclear correlations with biodiversity. Their performance is often strongly influenced by various non-biological factors, including recorder characteristics, background geophony (e.g., wind, rain, or river noise), and analytical design choices, i.e., decisions made during data processing and analysis that can substantially affect both results and interpretation [11,12,13,14,15].

Among these choices, the selection of specific eco-acoustic indices plays a critical role. Different indices respond variably to soundscape characteristics: some are more sensitive to insects, others to bird vocalizations, while some may be influenced by anthropogenic or abiotic sounds, leading to markedly different interpretations [11,16,17]. Indeed, temporal parameters such as the analysis window size and resolution, e.g., using 1 s vs. 1 min segments, affect data granularity. Short windows capture rapid, transient events but may increase noise sensitivity, whereas longer windows can smooth over variability that is typical of biotic signals [13]. Similarly, the selection of frequency bands (e.g., 2–8 kHz for birds) and threshold levels can either enhance detection of target species or inadvertently exclude significant acoustic activity. Misaligned frequency ranges may also overemphasize geophonic or anthropogenic sounds [18,19,20,21].

Noise filtering and pre-processing techniques, such as filters for wind or low-frequency background noise, while reducing environmental interference may also risk removing important biotic sounds, including specific amphibian calls or low-frequency mammal vocalizations [22]. Moreover, the use of different software and scripts for calculating eco-acoustic indices, such as Wavesurfer [23], Soundecology [24], MonitoR [25], and e.g. Seewave [26], involves varying default settings and processing algorithms. Key parameters like FFT window size, smoothing methods, and detection thresholds can significantly affect outcomes, even when using the same raw audio data [13].

In addition to these important factors, it has been shown that values and trends of eco-acoustic indices are not always comparable across studies. This is partly due to the wide range of commercially available recorders, which differ in cost, sensitivity, and frequency response, introducing biases and inconsistencies in index calculations [27]. These variations contribute to a broader lack of standardization in eco-acoustic research. As a result, the generalizability of acoustic indices as reliable proxies for biodiversity remains limited. This highlights the need for calibration across different environments and validation against independent biodiversity measures.

Researchers are increasingly urged to combine multiple metrics [28] or use composite indices tailored to each study area [29], either through statistical methods or by analyzing the autocorrelation and long-term memory of environmental sounds. Efforts to develop a universal index, such as the Soundscape Ranking Index, are still ongoing. Preliminary results aimed at providing an overall assessment of an area’s soundscape are promising, although further research is needed to refine these approaches [30,31].

At the same time, PAM deployments are expanding from short-term recordings to thousands of hours of audio data. This growing volume has renewed interest in simple descriptors that can be extracted quickly and compared across sites. For instance, the equivalent continuous sound pressure level, Leq, a widely used and standardized acoustic metric, meets this need: it can be calculated at any temporal resolution and, when using calibrated devices, can be directly compared to regulatory noise limits. Furthermore, analyzing the temporal dynamics of short-window Leq values (e.g., Leq_1s_) allows the identification of diel cycles in biophonic activity and anthropogenic disturbances, without requiring complex spectral processing. Preliminary unsupervised analyses have already demonstrated that peak-based metrics can effectively distinguish functional periods of bird, insect, and anthropogenic activity in urban soundscapes [18,32,33].

In this work, we study a highway-bisected woodland within the Ticino River Regional Park (northern Italy), an ecologically valuable area shaped by both natural processes and anthropogenic pressures. Using passive acoustic monitoring (PAM) recordings collected at multiple locations, we aim at: (1) computing the equivalent continuous sound pressure level at one-second resolution, Leq_1s_, (2) extracting inter-peak intervals as a proxy for vocal activity rhythms, and (3) assessing whether these simple, energy-based metrics can effectively capture soundscape structure. However, the complexity of overlapping acoustic sources often requires more nuanced analysis.

Our main goal consists in developing a methodological approach expected to enhance our understanding of a complex soundscape dynamics based on wavelet analysis [34], referred to as the maximal-overlap discrete-wavelet transform (MODWT) [35]. The method is built upon a decomposition of each signal into a series of frequency-resolved components which, retaining the original time resolution, turns out to be particularly well-suited for analyzing complex environmental recordings. Although  this study is grounded in an ecological context, its primary contribution is indeed methodological. We evaluate whether a wavelet-based decomposition using the maximal-overlap discrete wavelet transform (MODWT) can enhance the extraction and interpretation of simple energy-based acoustic descriptors (Leq_1s_, peak intervals) that are widely used in soundscape ecology, with the intent to help quantify ecological disturbance. Thus, the ecological differences described in this study are site-specific exploratory observations, being the MODWT analysis the primary methodological contribution.

We compute the sound pressure level and peak statistics from both the full-band signal and its frequency-resolved wavelet components. In addition, we calculate the autocorrelation function (ACF) of the wavelet components to estimate their respective decay times. Further validations are provided by an expert listening the audio files to quantify the presence and relative contribution of different sound sources. We expect that decomposing acoustic energy across wavelet levels will reveal distinct temporal signatures of biophonic, geophonic, and anthropogenic sources, thereby improving the robustness of simple eco-acoustic indicators.

The paper is organized as follows. In Section 2, we describe the area of study within the Ticino River Regional Park, the recording setup, how do we detect peaks within Leq_1s_ time series, and the wavelet decompositon used. Details of the validation procedure performed by an acoustic expert are discussed. In Section 3 we present the results covering the wavelet decompostion and filter used, peak distributions, Leq_1h_ as a function of hour of the day, and autocorrelation function of the Leq_1s_ time series. Section 4 is devoted to a discussion of the results and the concluding remarks.

## 2. Materials and Methods

We describe the study area, recording setup, energy normalization procedure, wavelet decomposition, autocorrelation decay-time computation, and expert annotation process.

### 2.1. Study Area

**Figure 1:**
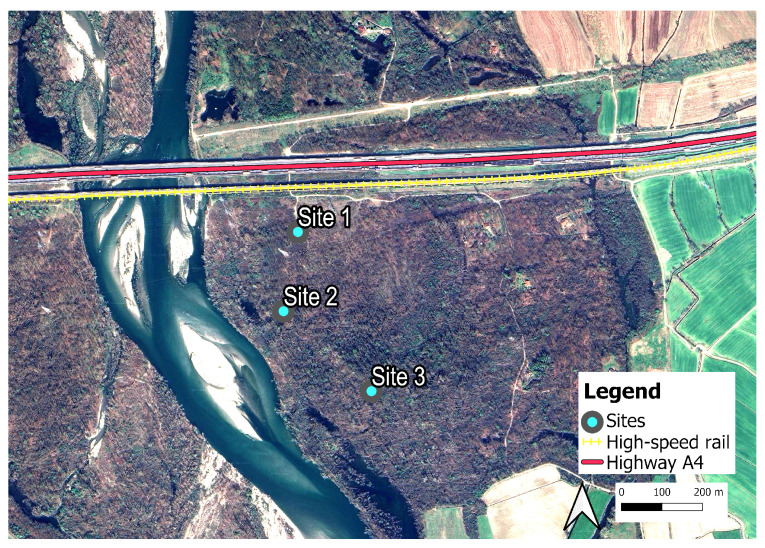
Study area with indications of the three monitoring sites. The study was conducted near Bernate Ticino (45°27′ N, 8°48′ E), located in the western part of Lombardy, approximately 30 km west of Milan. Highlighted are the high-speed railway and the A4 highway.

The Ticino River Regional Park is a protected area that includes extensive forests, wetlands, and meadows along the river. These riparian woodlands play a vital ecological role by supporting high biodiversity and ensuring habitat connectivity; however, they are particularly vulnerable to fragmentation caused by infrastructure such as roads and highways (Figure 1). Three monitoring sites were selected: Site 1, Site 2, and Site 3, situated approximately 100 m, 300 m, and 500 m, respectively, from the main technophonic noise sources (a highway and a high-speed railway).

### 2.2. Recording Setup and Data Collection

Acoustic data were collected between 26 May 2021 and 10 June 2021 using two Soundscape Explorer–Terrestrial (SET, Lunilettronik, Fivizzano MS, Italy) devices. Each device was equipped with environmental sensors for measuring: humidity, temperature, light, and atmospheric pressure; and two microphones: one optimized for low-frequency sounds (up to 48 kHz) and the other for high-frequency sounds (up to 192 kHz). Although each SET recorder includes two microphones (one low-frequency and one high-frequency), all analyses in this study were based on the recordings from the low-frequency microphone channel, sampled at 48 kHz with 16-bit resolution. This bandwidth (0–24 kHz) fully covers the range of interest for both biophonic and anthropogenic components while ensuring homogeneous data across all sites. The SET units were mounted approximately 4 meters above ground level on trees positioned along a transect oriented perpendicular to the main sources of anthropogenic noise in the area–namely, the A4 highway and a high-speed railway (see Figure 1).

Three sites were selected for monitoring, with each site observed for approximately one week. Due to the availability of only two SET devices, a sequential monitoring scheme was adopted. Initially, the devices were deployed at Site 1 and Site 3. After one week, the  device at Site 1 was relocated to Site 2. As a result, data collection was divided into two continuous monitoring periods:

- **Period 1:** From 26 May 2021 (13:00) to 2 June 2021 (23:54), recordings were collected from Site 1 and Site 3.
- **Period 2:** From 3 June 2021 (13:00) to 10 June 2021 (23:54), recordings were collected from Site 2 and Site 3.

Site 3 was intentionally kept active during both periods to serve as a clock reference, making possible the comparison between the two monitoring weeks data, and controlling for day-to-day environmental variability (e.g., weather or diel patterns). In contrast, Site 1 and Site 2 were alternated to capture the spatial gradient of anthropogenic disturbance, with Site 1 being closer to the highway and Site 2 located at an intermediate distance. Recordings were made at a 48 kHz sampling rate with 16–bit resolution, stored in WAV format. The recording schedule followed a duty cycle of 1 min of recording followed by a 5 min pause, resulting in 10 recordings per hour at each site. For simplicity, we selected just one day per site, and specifically, 1 June for Site 1, and 8 June for Site 2 and Site 3.

### 2.3. Peak Detection

For each 1 s interval, we computed the equivalent continuous sound pressure level as follows,

(1) $${\mathrm{Leq}}_{1s}=10\log_{10}\left(\langle p^{2}\rangle /p_{0}^{2}\right),$$

where $\langle p^{2}\rangle$ is the mean squared sound pressure over 1 s interval, and $p_{0}=20$ μPa is the reference pressure. Peaks were detected using the Findpeaks function from the Pracma package in R (version 2025.09.1) [36]. This procedure was applied identically to both the full-band signal and each wavelet component, enabling consistent multi-scale comparison of temporal soundscape patterns. More generally, we often use the standard definition,

(2) $${\mathrm{Leq}}_{\tau }=10\;\log_{10}\left(\frac{1}{\tau }\int_{t_{1}}^{t_{2}}dt\;\;\frac{p^{2}\left(t\right)}{p_{0}^{2}}\right),\;\tau =t_{2}-t_{1},$$

and depending on the problem considered, we take τ=1 s, 1 min or 1 h.

The Leq time series allows for the extraction of inter-peak intervals, or inter-peak lags, representing the time interval between two consecutive peaks in the Leq_1s_ time series, and the median inter-peak lag corresponds to the median of all such intervals calculated over each recording and wavelet level, minimizing the influence of outliers.

The energy normalization of the signal and each wavelet component is discussed in Appendix A. The wavelet decomposition used here is summarized in Appendix B, and the MODWT frequency bands are discussed in Appendix C. We proceed with a brief discussion on the type of wavelet filters employed.

### 2.4. Choice of Wavelet Filters

The performance of MODWT in analyzing acoustic signals depends on the choice of wavelet filter. Here, we evaluated three wavelet bases commonly used in environmental and bioacoustic applications: Daubechies-4 (d4), Daubechies-8 (d8), and Symlet-8 (or Least Asymmetric 8, la8). These filters differ in terms of interpolating polynomial order, symmetry, and time–frequency resolution—all of which influence their ability to capture specific spectral and temporal features of soundscapes.

Daubechies wavelets [37,38,39,40,41,42] form a family widely used in signal processing. Each wavelet is identified by its order *N* (e.g., d4, d8), which determines two key properties: the order of the interpolating polynomial (also known as the number of vanishing moments) and the filter length (equal to 2N taps). The filter length governs the time–frequency tradeoff: longer filters offer better frequency resolution but poorer time resolution, and vice versa.

Daubechies wavelets are particularly effective for detecting sharp acoustic events or filtering out background trends. Symlet (la8) wavelets, introduced as a modification of the Daubechies family, retain similar properties but are designed to be nearly symmetric. For example, la8 has the same number of vanishing moments as d8, but  introduces less phase distortion. This makes Symlets especially useful in applications where preserving waveform shape and timing is important.

In our context of soundscape monitoring, we tested three wavelet filters which may offer distinct advantages:

- d4: Offers better time localization and is suited to detecting short transients, chirps, and sharp onsets in birdsong or anthropogenic pulses.
- d8: Provides better frequency resolution and is more effective at capturing harmonic or tonal components such as whistles or environmental hums.
- la8: A modified version of d8 with near symmetry, that can be useful to detect signals such as trills or insect calls.

### 2.5. Temporal Fractal Analysis

Fractals are structures characterized by scale-invariant patterns [43]. They provide a powerful framework for describing the complexity inherent in natural systems and have been applied across numerous disciplines [44,45], including ecology [46,47,48], which is particularly relevant to this study.

Fractal scaling offers an effective methodology for investigating acoustic complexity. The specific method used often depends on the nature of the signal being analyzed and the goals of the study [48]. In the fields of acoustics and music, several notable applications of fractal analysis have been reported [49,50,51]. However, fractal methods are generally more suitable for assessing the complexity of acoustic environments as a whole, rather than individual acoustic events. Recent studies have applied fractal analysis to estimate the fractal dimension of tropical acoustic communities and urban parks [52,53,54,55].

Here, we study the scaling behavior of time series derived from Leq values of both the full-band signal and its wavelet decomposition levels. Temporal scaling is quantified using the Hurst exponent, *H*, and several robust techniques are available for accurately estimating *H* [56,57]. Here, we use an indirect approach via the well-known relation,

(3) H=1−γ/2,

where γ is the scale-invariant exponent describing a possible power-law decay of the autocorrelation function (ACF) of the time series. We estimate γ by identifying the temporal interval over which the autocorrelation function (ACF) follows a power-law decay, typically observed at shorter time scales. Values of γ in the range 0<γ<1 correspond to 1>H>1/2, indicating scale-invariant persistent correlations and long time memory. Conversely, when γ>1, correlations are short-ranged, and the system behaves like a standard RW, with H=1/2.

In our context, we expect natural soundscapes, such as those dominated by soniferous species, to exhibit intrinsic complexity and long-range temporal correlations. As a final remark, the term long memory in this study refers to time series whose autocorrelations decay according to a power-law at short time scales.

### 2.6. Validation of Acoustic Analysis Through Listening-Based Annotation

To validate the automated analysis, we conducted a listening-based annotation of the recordings to quantify the presence of biological sounds, anthropogenic noise, and natural non-biological sounds at each site. For each one-minute recording selected from the representative days, an expert listener evaluated and annotated the following:

- Biological activity, primarily bird vocalizations. Bird numerosity: classified into three levels: none (value 0), few (value 1), many (value 2). Bird singing duration: classified as fraction of occupied singing time in each recording (range 0 to 1). Bird species: classified into none (value 0), one species (value 1), more than one (value 2). Bird distance: classified into none (value 0), close (value 1), far (value 2).  
- Anthropogenic noise, with a focus on traffic-related sounds. Traffic activity: categorized as none (value 0), continuous (value 1), or intermittent (value 2). Traffic distance: classified into none (value 0), close (value 1), far (value 2). Train presence: classified into none (value 0) and present (value 1).

Specifically, each categorical score (0, 1, 2) was linearly rescaled to the [0–1] interval for visualization purposes. The perceptual thresholds were defined through preliminary listening sessions to ensure consistent semi-quantitative classification across recordings: 0 = absent, 1 = intermittent < 30% of the minute, 2 = dominant > 30–40%.

The presence of natural non-biological sounds was not significant for the selected analyzed period. To ensure consistency and minimize variability related to individual hearing sensitivity, all recordings were annotated by a single expert listener. Multiple listening trials were conducted to establish clear annotation criteria and enhance the reliability of the perceptual assessment. This iterative process helped refine the identification of key features, leading to a more robust qualitative classification of soundscape components.

## 3. Results

For each site, the recordings were processed in hourly batches of 10 wav files. Each file was energy-normalized to a spectrogram-based reference before analysis. One-second Leq_1s_ values were then computed for both the broadband (full) signal and for 10 wavelet bands (W1–W10). Finally, peak intervals in the 1 s series were extracted to characterize the temporal distribution of high-energy acoustic events within each hour.

**Figure 2:**
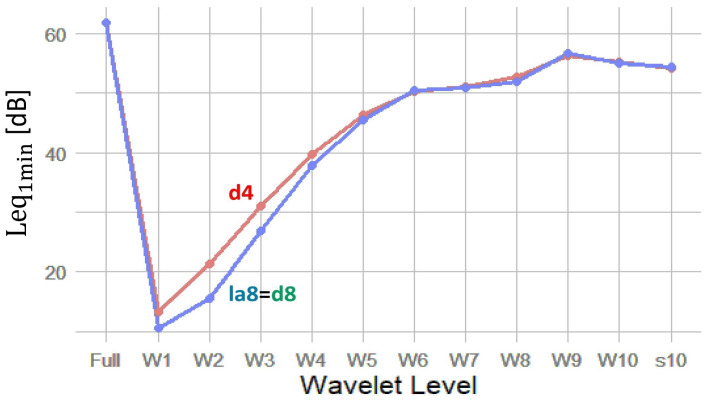
Leq_1min_ [dB] as a function of Wavelet Level for d4, d8, la8 filters, calculated for a typical recording of 1 min duration (Equation (2)). To be noted is that the results for d8 and la8 are indistinguishable at this resolution.

To select the most appropriate wavelet filter for our analysis, we compared Leq calculations using a representative recording from the study area. The results, presented in Figure 2, show Leq_1min_ values across wavelet levels for the d4, d8, and la8 filters.

As shown in Figure 2, the d8 and la8 filters produce nearly identical results across all wavelet decomposition levels. In contrast, the d4 filter enhances energy in the higher wavelet levels (W1–W4), which correspond to the mid-to-high frequency range (f>3 kHz). This range also includes non-biological components near the Nyquist limit, but since the frequency band (3–24 kHz) generally includes the most common biophonic components in woodland soundscapes—such as bird vocalizations and certain insect calls—we selected the d4 wavelet filter to better capture high-frequency biological activity. The d4 wavelet possess a superior temporal localization, allowing improved detection of short, transient acoustic events such as bird calls or anthropogenic pulses.

**Figure 3:**
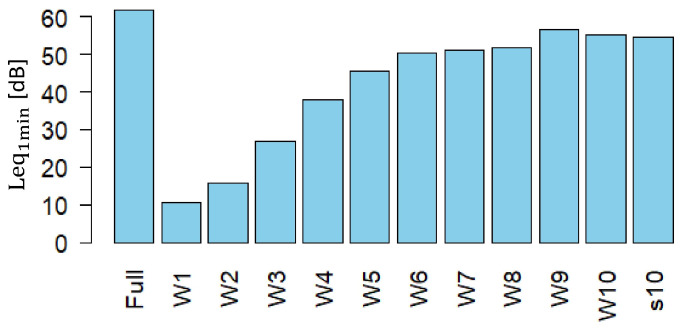
Leq_1min_ [dB] vs wavelet decomposition. Shown is the level distribution of energies among wavelets using d4 filter calculated for a typical recording (cf. Figure 2).

Before proceeding, we verified that the energy distribution across all the wavelet decomposition levels was consistent with the total energy of the original signal. An example of this distribution, calculated using the d4 filter for the same recording, is shown in Figure 3. The figure shows that most of the acoustic energy is concentrated in the set of levels (W6–W10), which correspond to the mid-to-low frequency range.

**Figure 4:**
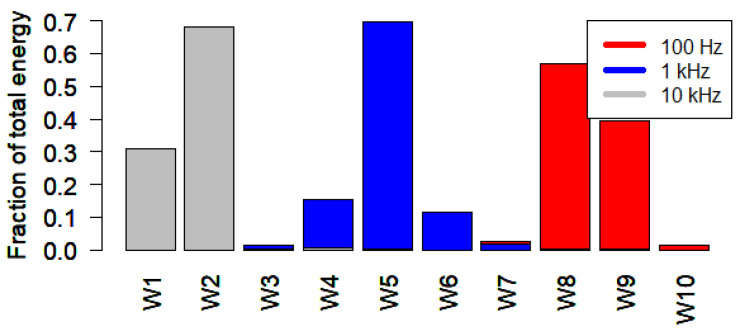
Fraction of total energy distribution across wavelet levels for three pure tones (100 Hz, 1 kHz, 10 kHz) using d4 filter and MODWT in R.

We also computed and compared the fractional wavelet energy distribution across levels for three pure tones (100 Hz, 1 kHz, and 10 kHz) using MODWT in R. The results, shown in Figure 4, illustrate that lower frequencies (e.g., 100 Hz) concentrate more energy in higher wavelet levels (such as W10 and W9), while 1 kHz maps to mid-levels (around W5), and higher frequencies (e.g., 10 kHz) are represented in the lower wavelet levels (W1 and W2). These results confirm the frequency localization capability of wavelet decomposition.

To investigate temporal patterns in acoustic activity, we computed the equivalent continuous sound pressure level at 1 s resolution, Leq_1s_, for all recordings across the three sites. From these time series, we extracted peaks as proxies for sound events, distinguishing between two detection modes: (1–1) peaks, which require a single decrease on either side of the peak, and (2–2) peaks, which require at least two consecutive decreases on both sides. While the (1–1) mode captures rapid fluctuations and short-lived events, the (2–2) mode acts as a low-pass filter, emphasizing more prominent and structured events.

**Figure 5:**
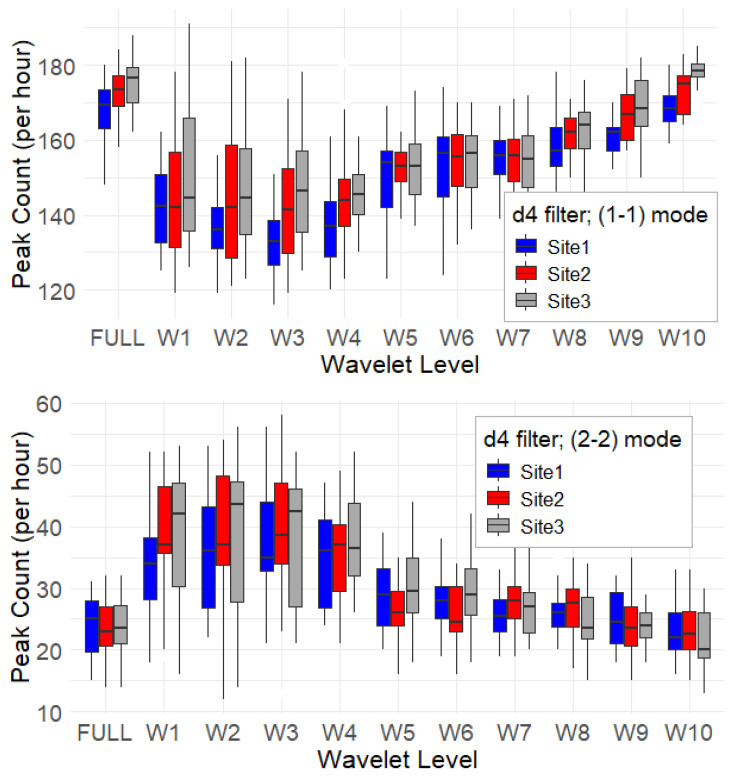
Hourly peak count rate as a function of wavelet level for the (1–1) and (2–2) modes using the d4 filter, for the three sites.

The intervals between successive peaks were then used to estimate sound-activity dynamics, providing insight into the typical temporal spacing between acoustic events. Figure 5 shows the hourly peak count rate by wavelet level for both (1–1) and (2–2) modes, using the d4 filter. Overall, the peak counts for the (1–1) and (2–2) modes differ significantly, confirming that the (2–2) mode is, as expected, more selective. Additionally, the two modes exhibit opposite trends across wavelet decomposition levels. The (1–1) mode shows lower peak counts in the lower decomposition levels (W1–W4), with values steadily increasing toward higher levels. Within this pattern, Site 3 consistently shows higher peak counts, particularly in (W1–W4) and (W8–W10). In contrast, the (2–2) mode yields higher peak counts in the lower decomposition levels and an almost flat trend across the remaining levels. In this case as well, Site 3 shows the highest peak counts for (W1–W3), followed by Site 2.

**Figure 6:**
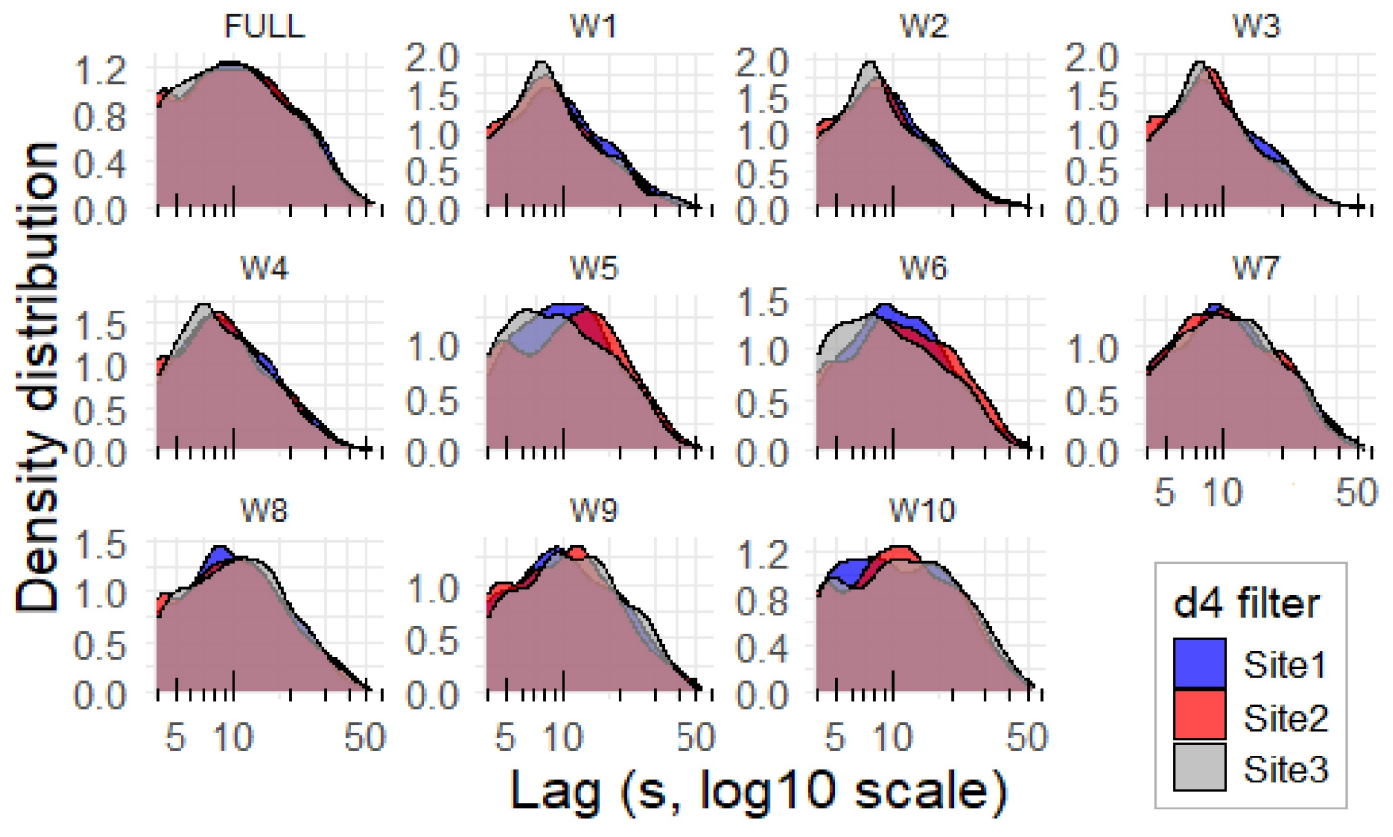
Distribution of inter-peak lags calculated for the (2–2) mode using the d4 filter.

Figure 6 shows the density distribution of inter-peak lags (see Section 2.3) calculated for the (2–2) mode using the d4 filter. In contrast, the (1–1) mode did not reveal any substantial differences. In Figure 6, noticeable variations are observed mainly for wavelet decomposition levels W5, W6, and W10.

As shown in Table 1, the median inter-peak lag is consistently around 10 s across all sites and wavelet levels, indicating a relatively stable underlying pattern of sound activity. However, Site 3 generally exhibits shorter mean lags in the mid-frequency wavelet levels (W2–W6), suggesting denser acoustic activity in these bands. For instance, at level W5, Site 3 has a mean lag of 11.8 s and a median lag of 9 s, compared to 10 s for both Site 1 and Site 2. Similarly, at W6, Site 3 shows a mean lag of 11.7 s and a median lag of 9 s, while Site 2 records a longer mean of 12.7 s and a median of 11 s. These shorter lag values indicate more frequent peaks (i.e., shorter intervals between sound events), which may reflect either higher sound activity or more tightly clustered acoustic events at Site 3 in those frequency bands. Furthermore, Site 3 consistently shows a higher number of peaks in the lower wavelet levels, e.g., (W2–W4), reinforcing the interpretation of denser acoustic activity. For example, Site 3 records 876 peaks at W2 and 916 peaks at W3, which align with the observed shorter lags. This increased activity may point to specific species or sound sources that are more active or acoustically dominant in that frequency range.

**Figure 7:**
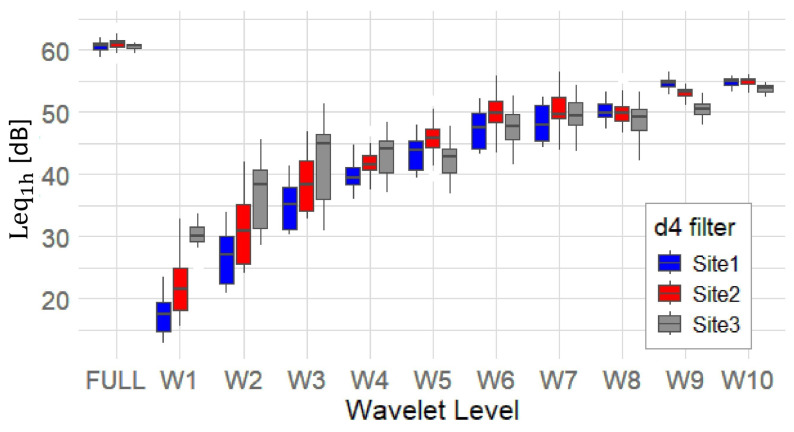
Leq_1h_ [dB] distribution vs. wavelet level, for each site, using d4 filter. The boxplots display median (central line), quartile and outlier information (bars) for each wavelet level and site.

Figure 7 shows the hourly Leq_1h_ distribution by wavelet level and site using the d4 filter. The FULL (original signal) band exhibits the highest Leq (≃ 60 dB) across all three sites, while W1 and W2 have the lowest. The increase in Leq from W1 to W5 indicates a general upward trend, peaking around (W5–W7), implying that mid-frequency bands dominate the acoustic energy at all sites. Site 1 and Site 2 have lower W1/W2 medians than Site 3, suggesting less energetic high-frequency content.

Between W7 and W10, Leq values plateau, suggesting that low-frequency components are relatively consistent in energy across sites. Site 3 generally shows slightly higher Leq values at lower levels (W1–W4), possibly indicating more high-frequency or impulsive events (e.g., insect calls), while Site 2 has higher median values at (W5–W7), reflecting persistent mid-frequency sounds. At higher levels (W8–W10), differences between sites converge, though Site 1 maintains a higher median value, likely due to greater exposure to technophonic sound sources.

**Figure 8:**
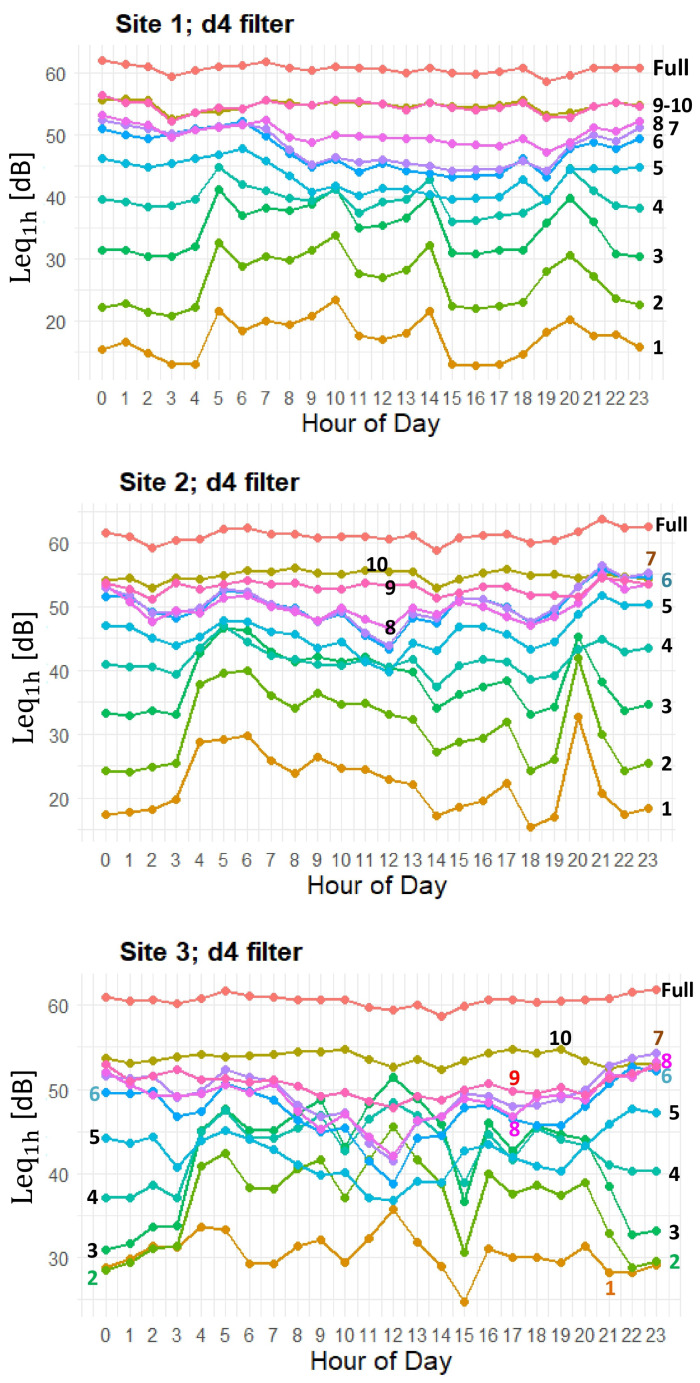
Leq_1h_ [dB] values vs. hour of the day, for W1–W10 and full signal, using d4 filter.

Figure 8 illustrates the hourly evolution of Leq values across wavelet levels for Site 1, Site 2, and Site 3, respectively, using the d4 filter. The lines represent the mean Leq at each hour for the full signal (FULL) as well as for individual wavelet levels (W1–W10). At all sites, the FULL signal remains elevated throughout the day, averaging around 60 dB, with minor fluctuations that are difficult to discern due to the axis scale. In contrast, the wavelet-decomposed levels exhibit more dynamic behavior, particularly at finer temporal scales. For example, levels (W1–W4) display pronounced diurnal variations, whereas higher-level components (W8–W10) are comparatively stable.

Site 1 shows a well-defined diurnal rhythm. The finest level (W1) presents a clear midday trough, with higher values at dusk and in early morning. Similarly, though less pronounced, patterns appear in the mid-frequency bands (W3–W6), with Leq values typically decreasing from late morning to early afternoon before rising again in the evening. The low-frequency levels (W9–W10) remain relatively flat, indicating more stationary contributions at those scales.

At Site 2, the pattern is more irregular than for Site 1. Although a midday decrease is evident in many bands, the finest levels (W1–W3) occasionally show sudden increases, most notably around 04:00 and 19:00—spikes that are less prominent at Site 1. The mid-frequency bands (W4–W6) follow a broadly similar trend to Site 1 but with greater variability. As with Site 1, W9 and W10 remain relatively constant over the day, though slight evening increases are observable.

Site 3 displays yet a more complex and irregular pattern than for the other two sites. For higher frequencies (W1–W4), Leq values are both higher and more variable. Notably, (W1–W4) exhibit a dip at 15:00, displaying abrupt changes around 03:00 and 10:00. The mid-frequency bands are noisier than at the other sites, and unlike the Site 1 smooth profiles, Site 3 fluctuations are more erratic. Once again, W9 and W10 remain relatively flat, with minor evening increases, as observed at Site 2.

We now present the results of our empirical analysis of temporal scaling in both the original signal and its wavelet decomposition. The aim is to quantify the possible presence of a power-law correction to the temporal decay of the ACF of the broadband Leq_1s_ time series for the three sites, described by the relation.

(4) $$y\left(t\right)=y_{0}\;\;t^{-\gamma }\;\exp\left(-t/\beta \right),$$

Equation (4) containing three fitting parameters, $y_{0}$, γ and β, with the idea of making contact with the fractal analysis discussed in Section 2.5. To this end, we attempt to estimate the exponent γ using Equation (4) from the time series derived from both the full broadband signal and wavelet-decomposed (W1–W10) Leq representations. As outlined in Section 2.5, ACFs were computed for each site and wavelet component over a 24 h period.

**Figure 9:**
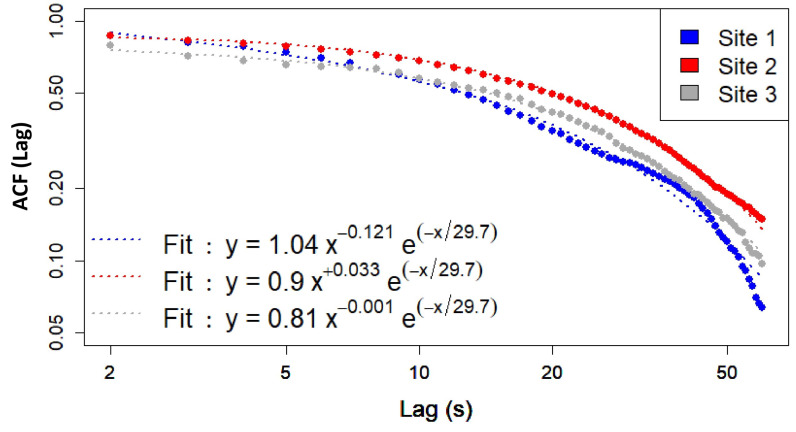
ACF of Leq_1s_ time-series for: Site 1 (blue circles), Site 2 (red circles) and Site 3 (gray circles). The fits using two parameters, $y_{0}$ and γ are reported in the inset (dashed lines).

**Figure 10:**
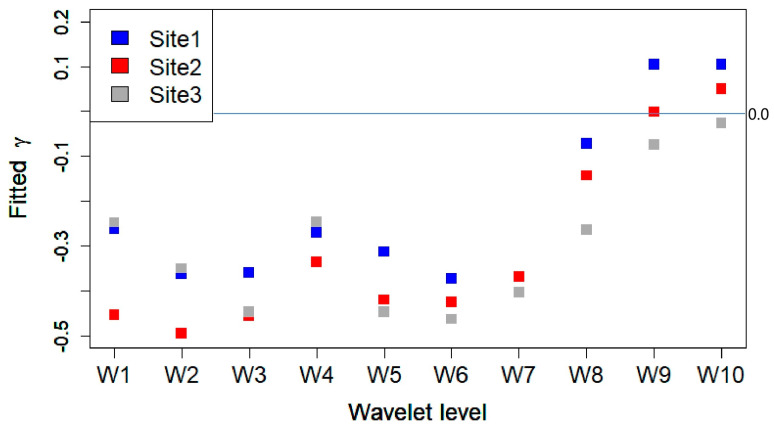
Fitted γ across all wavelet decomposition levels and for each site. For W7, γ coincides for Site 1 and Site 2. The horizontal line corresponds to γ=0, and only positive values can be interpreted using Equation (3). Additional measurements are required to improve on the temporal behavior of the ACF.

We first perform a fit with the three parameters for each site, and evaluate the mean value 〈β〉 over the three sites. By keeping β=〈β〉≃29.7 fixed in (4), thus reducing the number of fit parameters to two, we obtain new fits for $y_{0}$ and γ, displaying an accurate behavior. Note that we do not impose the constraint that γ>0 for performing the fit, so that negative values of γ can eventually occur. In those circumstances, our approach based on Equation (3) does not apply (see below). As shown in Figure 9, Site 1 exhibits the steepest decay (γ≈0.121), while Site 2 and Site 3 show flatter decays (γ≃−0.033≈0 and γ≈0.001, respectively), indicating slightly different sound dynamics between Site 1 and (Site 2–Site 3) at short time scales. These results are only preliminary, and larger time series are needed before drawing general conclusions on the behavior of the ACF. Figure 10 shows the fitted γ values for each wavelet decomposition level (W1–W10) and site.

The results in Figure 10 reveal a strong frequency dependence in temporal scaling behavior: high-frequency components (W1–W8) have negative γ values, whereas the low-frequency ones (W9–W10) yield positive 0≲γ≪1, consistent with a conspicuous persistence described by anomalous diffusion exponents 1/2≪H≲1 (Equation (3)). Site-specific differences are evident across all levels, pointing to spatial variability in the temporal dynamics of the acoustic environment. The results suggest that the ACF for Site 1, which is closer to the highways, displays a faster time decay than for Site 2 and 3, as one may expect. Tentatively, we may interpret negative values of γ, found for the high-frequency wavelets, as representing bird sound activity almost unaffected by the relatively low-frequency disturbances produced by road and railroad traffic.

## 4. Discussion and Concluding Remarks

This study is experimental in nature, based on a limited dataset composed of three sites and a single 24 h recording period at each location. As such, the ecological differences we report, such as higher biophonic activity at Site 3 or stronger anthropogenic signatures at Site 1, should be interpreted strictly as site-specific patterns rather than generalizable ecological trends. Our primary aim is not to draw broad ecological conclusions but to use these recordings as a controlled test bed for evaluating the performance of MODWT-based, frequency-resolved energy metrics and peak-interval analysis. Therefore, the ecological observations serve mainly as illustrative examples that demonstrate how the proposed methodology responds to real soundscape variability.

We utilize the MODWT to decompose soundscape audio into localized components. We selected the d4 wavelet filter because its shorter support provides better temporal resolution for detecting fast, transient events and enhances energy representation in higher frequency bands (W1–W4), which are typically rich in biophonic signals (e.g., bird and insect calls). While d8 and la8 produced comparable overall Leq distributions, they did suppress energy in these higher bands. The decomposition confirmed expected spectral localization: higher wavelet levels (W8–W10) captured low-frequency energy, which are likely dominated by abiotic sounds (wind, traffic), while lower levels (W1–W4) were sensitive to high-frequency components, typically associated with biophonic sources like bird and insect calls.

Short-term acoustic events were isolated using the peak-search modes ((1–1) and (2–2)) to differentiate transient from structured events. The (1–1) mode, sensitive to rapid fluctuations, showed a general increase in peaks toward lower frequencies, with Site 3 dominating, especially at (W1–W4) and (W8–W10). The (2–2) mode, which emphasizes structured events (due to its temporal smoothing), revealed distinctive activity patterns with Site 2 and Site 3 exhibiting higher peak counts. Inter-peak lag distributions (Figure 6 and Table 1) strongly supported denser acoustic activity at Site 3, particularly in the high-to-mid-frequency bands (W1–W6). Although all sites had a median lag near 10 s, Site 3’s shorter mean lags in these key bands suggest a more clustered vocal landscape, potentially indicating overlapping or competitive calling behavior.

Hourly Leq trends (Figure 7) revealed distinct site-specific differences. For instance, Site 3 consistently showed higher Leq in the high-frequency (W1–W4) levels, indicating a richer high-frequency biophony (e.g., birds, insects). Site 2 prevailed in the mid-frequency (W5–W7) range (likely technophony), while Site 1 exhibited higher Leq at the low-frequency (W8–W10) levels. Overall, Site 3 concentrated its maximal energy in the mid-to-low bands (W6–W10), reinforcing the presence of rich high-frequency biophony in (W1–W4).

The hourly Leq trends across all sites (Figure 8) reveal the diel rhythm of biological activity, primarily through dawn and dusk peaks in levels (W1–W6). The mid-day decline in acoustic energy is likely due to reduced vocalization, possibly from thermal or light constraints. Conversely, the flatter profiles observed in the lowest-frequency bands (W9 and W10) at all sites represent temporally stable background sounds or abiotic sources (e.g., geophonies like wind or distant traffic) with minimal hourly variation. These results validate MODWT-based Leq tracking as an effective method for capturing these diel acoustic signatures.

Site 1 shows a relatively smooth, structured diurnal pattern at finer wavelet levels, reflecting the natural ecological dynamics and predictable daily cycles of the biophonic activity. The proximity to the highway is evident in similar Leq trends in the low-frequency (W8–W10) decomposition levels. Site 2 also displays an almost regular pattern but with more pronounced peaks (around 04:00 and 19:00), suggesting transient or irregular sound sources, such as intense bird choruses, indicative of a more complex acoustic environment. Site 3 presents a more erratic pattern in the high-mid-frequency bands (W1–W5); its Leq profiles are not as smoothly modulated as Site 1’s. Crucially, the Leq levels in these bands are much higher than the other two sites, strongly suggesting the presence of closer and louder biophonies.

Temporal fractal analysis, describing the complexity and persistence of sounds, shows that Site 2 and Site 3 display similar persistence (up to rounding errors) in sound activity, followed by Site 1 (Figure 9). Lower values of the γ exponent (indicating a slower decay of the ACF) support this conclusion. Site 1, which is the closest to road and railroad traffic, is characterized by the fastest ACF decay.

A multiscale analysis of the numerically obtained γ values across wavelet levels (W1–W10) revealed a strong frequency dependence in temporal scaling (Figure 10). High-frequency levels (W1–W7) exhibited even negative γ values, suggesting that our approach based on Equation (3) no longer applies. We interpret this result by suggesting that high-frequency wavelet time series are unaffected by the low-frequency perturbations originated from the road and railroad traffic. In addition, site-specific differences in persistence were also consistent across all levels, reflecting spatial variability in the acoustic environment’s temporal dynamics.

**Figure 11:**
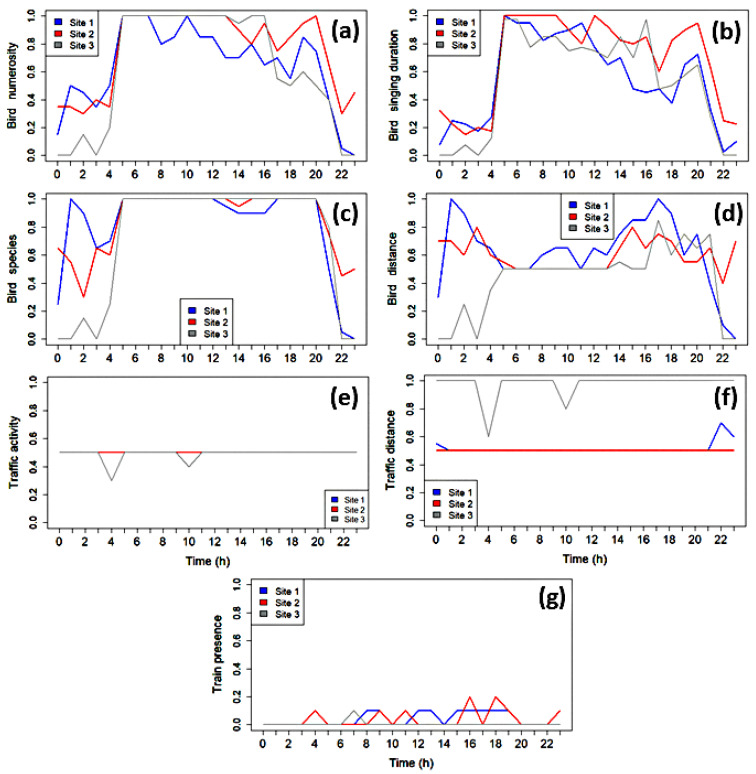
Expert-based annotations for: (**a**) bird numerosity, (**b**) bird singing duration, (**c**) bird species, (**d**) bird distance, (**e**) traffic activity, (**f**) traffic distance and (**g**) train presence, across 24 h at each site. Values are normalized to the [0, 1] range per variable defined in Section 2.6. We note that in (**e**) the results for Site 1 and Site 2 coincide.

To validate the MODWT-based spectral analysis, we performed extensive listening-based annotations on one-minute recordings from each site over the analyzed period (see Figure 11). It is found that bird activity and abundance are negatively related to proximity to anthropogenic disturbance. Bird abundance and songs were lowest at Site 1 (closest to disturbance) and highest at the more distant Site 2 and Site 3, suggesting birds are more vocally active where noise is less intense [18,58]. Species richness did not strongly correlate with distance, but Site 3 maintained the most stable species diversity. Spatially, birds were heard farther from the recorder at Site 1, suggesting avoidance behavior in noisier environments. Traffic intensity and train counts were also highest at Site 1. Overall, these findings support the hypothesis that anthropogenic noise negatively impacts bird activity, diversity, and spatial behavior, with the strongest effects near human infrastructure [6,59].

Expert manual annotations (Table 2) confirmed the automated MODWT findings. First, the duration and intensity of birdsong temporally aligned with peak Leq values in (W1–W4) and high peak rates, especially at Sites 2 and 3 during dawn/afternoon. Annotations also confirmed a higher biophonic presence at Site 3, validating its elevated wavelet-level energy and shorter inter-peak intervals. Second, traffic profiles confirmed site distinctions: Sites 1 and 2 have more significant traffic disturbance, while Site 3 is primarily affected by continuous, distant, low-frequency residual noise. This aligns with W9–W10 energy distributions and explains the flat Leq values at Site 2, suggesting some of its mid-band energy may originate from biophonic sources.

Overall, the convergence between manual annotations and automated metrics do reinforce the robustness of MODWT-based acoustic monitoring and validates its potential for long-term eco-acoustic assessment in multi-source soundscapes. Further in-depth analysis focusing on species and source recognition could be of help to better interpret these findings.

In summary, this study stresses the advantage of integrating simple energy-based metrics with multi-resolution wavelet analyses to characterize complex environmental soundscapes. By combining the equivalent continuous sound pressure level at one-second resolution (Leq_1s_), inter-peak interval statistics, and the maximal-overlap discrete-wavelet transform (MODWT), we were able to capture both the spectral and temporal dynamics of acoustic activity across a highway-bisected woodland in the Ticino River Park near Bernate, Italy. Our findings show that wavelet decomposition enhances the interpretability of Leq and peak-based metrics by isolating frequency-specific patterns of biophony, geophony, and technophony. Site-specific differences—such as the higher biophonic activity and vocal persistence observed at Site 3, or the spectral flattening and lower fractal persistence near the highway at Site 1—underscore the sensitivity of these methods to both ecological and anthropogenic influences. Hourly trends in wavelet-level Leq and autocorrelation metrics revealed diel periodicity and long-range temporal structures associated with biological activity, findings corroborated by expert listening.

The convergence between spectral analysis and expert annotation validates MODWT-based acoustic metrics as a suitable tool for eco-acoustic monitoring. Furthermore, the use of physically interpretable descriptors, such as Leq_1s_ and inter-peak intervals, provides a valuable complement to traditional eco-acoustic indices, particularly in heterogeneous, multi-source environments. As this study was based on a limited temporal dataset and only three monitoring sites, our results should be interpreted as a methodological validation rather than a full ecological assessment. Future work will focus on extending this approach to longer time series, additional habitats, and integration with machine-learning-based source identification to improve ecological interpretation.

Because the small dataset employed, consisting of only three sites and monitored over one day each, the ecological patterns identified in this study cannot be generalized beyond the specific sampling context. The conclusions with ecological relevance should therefore be considered exploratory. In contrast, the methodological findings, that is the ability of MODWT to reveal frequency-specific energy dynamics, enhance peak-based descriptors, and highlight temporal decay properties, are the core contribution of this work (see the comparison with traditional ecoacoustic indices discussed in Appendix C). The study should thus be viewed primarily as a proof-of-concept demonstration of a multi-resolution acoustic analysis framework, with ecological interpretation offered only as an application example.

This study also presents limitations in its experimental design, particularly regarding the non-simultaneous nature of the recordings. The  three sites were monitored on different days, which introduces potential variability especially related to weather. To mitigate these issues, we examined meteorological and physical data from the ARPA weather station in Magenta during the recording period. During the measurement days, no hourly average wind speed exceeded 5 m/s and no hourly average precipitation exceeded 2 mm/h, so no recordings required exclusion. While these checks confirm that weather conditions were broadly comparable and free from major disturbances, we acknowledge that a full analysis of temperature effects was not performed. These environmental variables can influence vocal activity and soundscape structure, and future studies using simultaneous or repeated sampling designs will be necessary to better control and quantify their role.

## Figures and Tables

**Table 1:** Summary of inter-peak lags statistics by Site (1st column) and Wavelet Level (2nd column). The mean 〈Lag〉 (3rd column) are expressed in [s]. The relative differences of 〈Lag〉 between sites, R [%] (4th column), are reported as follows: R_12_, R_13_, R_23_, and defined as: R = (<Lag>_larger_ − <Lag>_smaller_)/<Lag>_smaller_. The largest differences occur for W2: R_12_ =(11.5−10.0)/10.0=15%, and R_13_ =(11.5−10.2)/10.2=12.7%. We find <R> = 4.3% and $\sigma_{R}$ = 3.45%. The values in bold are larger than <R> + $\sigma_{R}≃7.8$%. Median lags [s] are reported in the 5th column. Median lag differences between sites, D [s] (6th column), are denoted as D_12_, D_13_, and D_23_, and defined as D*_ij_* = D*_i_* − D*_j_*. Non vanishing differences are highlighted in bold. The 7th column report the number of peaks found for each site and wavelet level.

Site	Level	<Lag>	R [%]	Median Lag	D [s]	N Peaks
Site1	FULL	12.7	2.4	10	0	551
Site2	FULL	12.4	0.8	10	0	533
Site3	FULL	12.6	1.6	10	0	550
Site1	W1	11.9	**8.2**	**10**	**1**	623
Site2	W1	11.0	**8.2**	**9**	**1**	710
Site3	W1	11.0	0.0	**9**	0	722
Site1	W2	11.5	**15.0**	**9**	**1**	722
Site2	W2	10.0	**12.7**	**8**	**1**	842
Site3	W2	10.2	2.0	**8**	0	876
Site1	W3	10.4	5.9	8	0	875
Site2	W3	9.82	4.0	8	0	888
Site3	W3	10.0	1.8	8	0	916
Site1	W4	10.7	1.0	**9**	0	829
Site2	W4	10.6	3.9	**9**	**1**	833
Site3	W4	10.3	2.9	**8**	**1**	877
Site1	W5	12.1	1.7	**10**	0	641
Site2	W5	12.3	2.5	**10**	**1**	592
Site3	W5	11.8	4.2	**9**	**1**	713
Site1	W6	12.3	3.3	**10**	**−1**	653
Site2	W6	12.7	5.1	**11**	**1**	611
Site3	W6	11.7	**8.5**	**9**	**2**	638
Site1	W7	12.8	5.8	10	0	570
Site2	W7	12.1	0.8	10	0	641
Site3	W7	12.7	5.0	10	0	622
Site1	W8	12.4	2.4	10	0	599
Site2	W8	12.7	4.8	10	0	601
Site3	W8	13.0	2.4	10	0	563
Site1	W9	12.4	0.0	10	0	582
Site2	W9	12.4	2.4	10	0	540
Site3	W9	12.7	2.4	10	0	559
Site1	W10	13.2	6.6	**10**	0	524
Site2	W10	12.4	3.0	**10**	**−1**	553
Site3	W10	13.6	**9.8**	**11**	**−1**	504

**Table 2:** Summary of bird and environmental metrics across a disturbance gradient: Bird numerosity (abundance), bird singing (duration), bird species (diversity). Arrows represent the direction of change from Site 1 to Site 3.

Variable	Site 1	Site 2	Site 3	Ecological Implication
	**(Near)**	**(Mid)**	**(Far)**	
Bird numerosity	↓	↑	↑	Birds avoid areas near high disturbance
Bird singing duration	↓	↑	↑	Higher vocal activity at quieter sites
Bird species	→	→	→	Little influence of anthropogenic noise
Bird spatial distance	Farther	→	Closer	Birds farther from recorder near disturbance
Traffic/trains	↑	↑	↓	Confirms proximity to roads/railways

**Figure A1:**
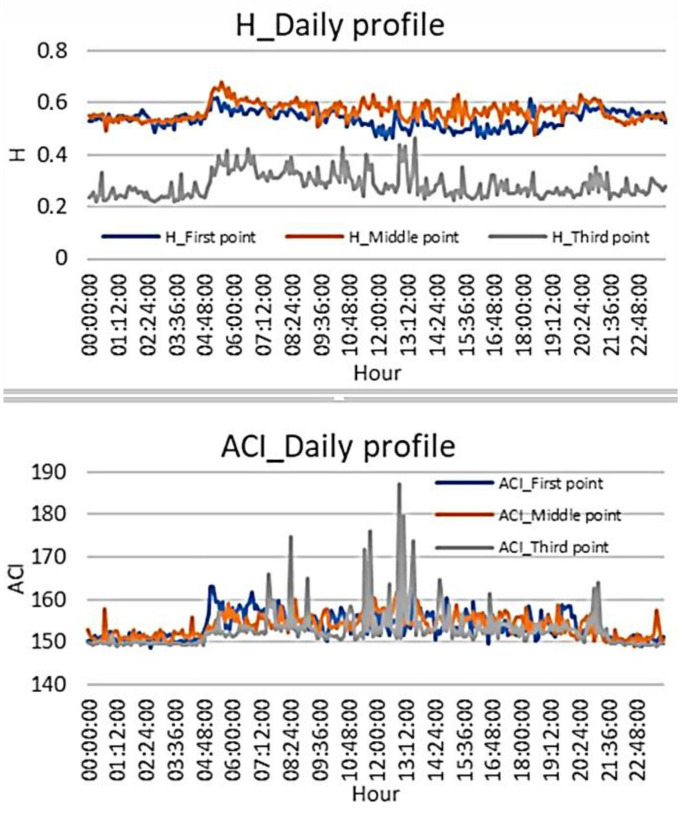
Conventional ecoacoustic indices, ACI and H, vs, hour of day.

## Data Availability

Data used in this study are available upon request.

## Appendix A. Energy Normalization

The energy normalization ensures the comparison of the full-band signal (original recording) and each wavelet component. The  full-band signal is first analyzed using a short-time Fourier transform (STFT) to derive a time-frequency representation of sound pressure levels. The reference energy is computed from the spectrogram as follows: First, the spectrogram amplitude is converted from dB to squared pressure,

(A1) $$p^{2}\left(f,t\right)=p_{0}^{2}\cdot 10^{A_{\mathrm{dB}}\left(f,t\right)/10},$$

where $A_{\mathrm{dB}}\left(f,t\right)$ is the spectrogram amplitude in dB. Then, the average squared pressure across all frequencies at time frame *t* is computed as

(A2) $$\langle p^{2}\left(t\right)\rangle =\frac{1}{F}\sum_{f=1}^{F}p^{2}\left(f,t\right).$$

Finally, a reference energy (proportional to Pa^2^) is obtained by averaging over time,

(A3) $$E_{\mathrm{ref}}=\frac{1}{T}\sum_{t=1}^{T}\langle p^{2}\left(t\right)\rangle ,$$

where *F* is the number of frequency bins (in our case we use 1024 FFT points) and *T* is the number of time frames. The  reference energy, $E_{\mathrm{ref}}$, serves as a common baseline for normalizing both the full waveform and its wavelet components.

Now, to ensure consistency between spectral and time-domain representations, the full-band signal is rescaled to match the reference energy obtained from the spectrogram. To do this, we first compute the average energy of the original audio signal,

(A4) $$E_{\mathrm{signal}}=\frac{1}{N}\sum_{n=1}^{N}s^{2}\left[n\right],$$

where s[n] is the signal amplitude at time sample *n*, and *N* is the total number of samples. Next, we apply a scaling factor to the signal to match the baseline energy:

(A5) $$s^{\prime }\left[n\right]=s\left[n\right]\cdot \sqrt{\frac{E_{\mathrm{ref}}}{E_{\mathrm{signal}}}},$$

which allows the normalized waveform $s^{\prime }\left[n\right]$ to obey the sum rule,

(A6) $$\frac{1}{N}\sum_{n=1}^{N}s^{\prime 2}\left[n\right]=E_{\mathrm{ref}}.$$

## Appendix B. Wavelet Decomposition

We apply MODWT to the normalized signal $s^{\prime }\left[n\right]$. This transformation decomposes the input signal into *J* levels of wavelet coefficients, where each level corresponds to a specific frequency band. Specifically,

(A7) $$\mathrm{MODWT}\left(s^{\prime }\right)\Rightarrow \left\{w_{j}\left[n\right]\right\},\;j=1,\ldots ,J.$$

Here, $w_{j}\left[n\right]$ represents the wavelet coefficient at level *j* and time index *n*. Unlike the standard discrete wavelet transform (DWT) [60], which downsamples the signal, MODWT preserves the original sampling rate. That is, the number of samples N remains the same for all wavelet levels. This allows temporal alignment between the original signal and all its wavelet components. Each wavelet level captures signal fluctuations at a different frequency scale. We compute the average energy at each level *j* using,

(A8) $$E_{j}=\frac{1}{N}\sum_{n=1}^{N}w_{j}^{2}\left[n\right],$$

and therefore, the total energy across all levels in the decomposition is given by,

(A9) $$E_{\mathrm{MODWT}}=\sum_{j=1}^{J}E_{j}.$$

In general, the total MODWT energy, $E_{\mathrm{MODWT}}$, does not match the energy of the original signal due to the non-orthogonal nature of the MODWT filters, thus we apply a uniform scaling factor to all wavelet levels. This scaling adjusts each wavelet coefficient as follows:

(A10) $${\tilde{w}}_{j}\left[n\right]=w_{j}\left[n\right]\cdot \sqrt{\frac{E_{\mathrm{ref}}}{E_{\mathrm{MODWT}}}},$$

so that the square root ensures that energy (which scales quadratically with amplitude) is properly matched. After scaling, the energy of the transformed wavelet components equals the reference energy:

(A11) $$\sum_{j=1}^{J}\left(\frac{1}{N}\sum_{n=1}^{N}{\tilde{w}}_{j}^{2}\left[n\right]\right)=E_{\mathrm{ref}}.$$

This procedure allows for a comparison of Leq indicators for the full signal and their wavelet decompositions. For the latter, the levels were computed using the modwt function from the Wavelets package in R [61].

## Appendix C. MODWT Frequency Bands

We used 10 MODWT decomposition levels since this depth fully covers the ecologically relevant frequency range of our recordings given the 48 kHz sampling rate. In practice, 10 levels allow separation of:

- <250 Hz: low-frequency anthropogenic rumble and river noise (geophony/technophony).
- 0.25–2 kHz: mammal, amphibian, and low bird vocalizations.
- 2–8 kHz: dominant bird biophony.
- >8 kHz: insect stridulation and high-frequency cues.

For each level $W_{j}$, the frequency interval is given by $j\approx \left(2^{-(j+1)}f_{s},\;2^{-j}f_{s}\right)$ Hz, where $f_{s}=48$ kHz is the sampling rate. The approximate frequency bands are reported in Table A1.

**Table A1:** Approximate frequency bands for each MODWT level decomposition. For the last band, the precise value 23.4 was rounded to 24. For the residual signal, s_10_ ∈ (0–24) Hz.

Level	Frequency Band (Hz)
$W_{1}$	12,000–24,000
$W_{2}$	6000–12,000
$W_{3}$	3000–6000
$W_{4}$	1500–3000
$W_{5}$	750–1500
$W_{6}$	375–750
$W_{7}$	188–375
$W_{8}$	94–188
$W_{9}$	47–94
$W_{10}$	24–47

As mentioned in Appendix B, MODWT does not downsample the signal. Therefore, each wavelet coefficient series retains the same length as the input signal. As a result, the transform is redundant and not orthogonal, meaning that energy is not uniquely partitioned across the decomposition levels. However, this redundancy allows for precise temporal alignment across all wavelet levels.

To be noted is that no field calibration-tone or microphone sensitivity-correction have been applied here. Consequently, the reported Leq values should not be interpreted as absolute sound pressure levels. However, because the same devices and settings were used consistently across all sites, the results still remain suitable for comparing sound level variations, spectral energy distributions and wavelet decomposition levels between sites.

The MODWT-based metrics provide clearer frequency-specific temporal dynamics than the conventional ecoacoustic indices, examples of which are plotted in Figure A1. While indices such as ACI or H summarize broadband patterns, they are known to be sensitive to geophony and recording conditions and often compress complex signals into single-value descriptors. In contrast, the MODWT decomposition allows γ, Leq_1s_, and peak statistics to be examined separately within ecologically meaningful frequency bands, making it easier to identify whether slow-decaying autocorrelation originates from biophony (mid–high levels) or from low-frequency anthropogenic noise (high-level MODWT components W7–W10). This complements and in some cases improves the interpretability of traditional indices.
